# Combination of Chitosan, Tea Polyphenols, and Nisin on the Bacterial Inhibition and Quality Maintenance of Plant-Based Meat

**DOI:** 10.3390/foods11101524

**Published:** 2022-05-23

**Authors:** Zenghui Dai, Linna Han, Zhe Li, Mengqing Gu, Zhigang Xiao, Fei Lu

**Affiliations:** College of Grain Science and Technology, Shenyang Normal University, Shenyang 110034, China; dai2045556814@163.com (Z.D.); hanlinna0723@outlook.com (L.H.); lizhe_cad@163.com (Z.L.); gmq19990518@163.com (M.G.); zhigang_xiao@126.com (Z.X.)

**Keywords:** plant-based meat, biological preservatives, sensory characteristics, physicochemical properties, microbes

## Abstract

Plant-based meat products have gained attention in the food industry and with consumers. Plant-based meat products primarily comprise plant proteins and are rich in nutrients. However, the products are highly susceptible to bacterial contamination during storage. Biological preservatives are easily degradable alternatives to chemical preservatives and can preserve different kinds of food. In order to investigate the preservation properties of chitosan (CS), tea polyphenols (TPs), and nisin treatments on plant-based meats, the sensory evaluation, color difference, pH, TBARS, and the total plate count of *E. coli*, *S. aureus*, and *Salmonella*, indicators of the biological preservative-treated plant-based meat, were determined in this study. The experiment involved blank control- and biological preservative-treated samples. We found that the total microbial count exceeded the national standard provisions in the control samples stored for 14 days. The colors, tissue structures, and flavors of plant-based meat have gradually deteriorated, with the sensory score dropping from 90 to 52. The sample had a loose tissue structure and an obvious sour taste. However, the shelf life of the plant-based meat samples treated with different combinations of the biological preservatives increased compared to the shelf life of the control samples. After 56 d of storage, 1% chitosan, 2.5% tea polyphenols, and 0.04% nisin sensory reduction to 56, the total number of colonies and *S. aureus* were 4.91 and 2.95 lg CFU/g, approaching the national standard threshold; *E. coli* was 2 lg CFU/g, reaching the national standard threshold. Thus, the samples treated with 1% chitosan, 2.5% tea polyphenols, and 0.04% nisin had the longest shelf life (56 days) among all experimental groups. Hence, this study reveals that a combination of biological preservatives may be a non-toxic alternative for the efficient preservation of plant-based meat products.

## 1. Introduction

With the rapid development of the social economy, people have become more health-conscious and are opting for healthier diets. Plant-based meat products using plants as raw materials are well received by consumers and are gaining attention in food research and development [1]. Plant-based meats, also known as vegetarian meat, simulated meat, and plant meat, primarily take plant protein, fat, and plant raw materials extracted from soybean, pea, wheat, and other crops. After extrusion, electrostatic spinning, and 3D printing, the new products resemble the texture and taste of animal meat products [2,3,4,5,6]. Extensive research has recently been conducted on plant-based meat [7,8,9,10,11,12,13,14,15,16,17,18,19]. Studies have investigated numerous properties of plant-based meat products, including the effects of water, protein, fat, seasoning agent, binder, and coloring agents on the flavor, color, texture, and nutritional value of the final product [7,8,9]; plant-based meat extrusion, cutting, and spinning methods [10,11,12,13]; and the ecology, economic demand, purchase, food safety, and nutrition of plant-based meats [14,15,16]. However, there are few reports about plant-based meat preservation. Plant-based meats with high moisture content are susceptible to bacteria, such as *Escherichia coli*, *Salmonella*, and *Staphylococcus aureus*, during their storage, thereby reducing their shelf life [20,21,22]. Geeraerts et al. [20] evaluated the composition of the microbial colonies of several ready-to-eat meat alternatives (including vegetarian, vegan meat imitations, and an insect sample) in the Belgian market, and found that vegetarian products mainly consisted of *Lactobacillus sakei*; isolates of vegetarian products mainly consisted of *Lactobacillus sakei*; isolates of vegan products were mainly composed of *Lb. sakei*, *Enterococcus faecium*, and *Carnobacterium divergens*; isolate insect products mainly contained *E. faecium*, *Macrococcus caseolyticus*, and *Cronobacter sakazakii*. Plant-based meat products remain fresh under chilled conditions for several days, which can be extended to a few weeks when they are packed in a modified atmosphere or have undergone post-packaging pasteurization [23,24]. Therefore, it is crucial to investigate effective preservation techniques for plant-based meat products.

Biological preservatives are gaining more attention. Natural agents were obtained from animals, plants, microorganisms, and bioengineering transformations. Most of them are non-toxic, biodegradable, and can be used on different kinds of food [22]. Using natural and efficient inhibitors and preservatives instead of traditional chemical inhibitors and preservatives has become a new trend. Chitosan is a biodegradable natural polysaccharide and the only alkaline glycan present in nature [25]. It is non-toxic, degradable, has a film formation ability, and has a significant inhibiting effect on common food bacteria, fungi, and yeasts [25]. Hence, chitosan is used to preserve fruits, vegetables, meat, and aquatic organisms [25,26]. Chitosan and gelatin are normally used as edible preservation coatings for food. For example, in a previous study, compared to uncoated and gelatin-coated beef, chitosan coating significantly reduced fat oxidation and lactic acid bacterial growth [27]. Furthermore, when frozen pork was preserved with a eugenol nanocapsule incorporated into gelatin/chitosan [28], the storage period was extended by 7 days, and the preservative effectively controlled the increase in the pH, total volatile salt-based nitrogen (TVB-N), and thiobarbituric acid-reactive substances (TBARs). Another study [29] added chitosan as a coagulant, which increased the shelf life of chitosan tofu by 3 days compared to when calcium chloride was added. Tea polyphenols are other biological preservatives that are non-toxic, have excellent antioxidants, and inhibit most food-borne bacteria growth [30]. Tea polyphenols are used in meat and aquatic products, fruits and vegetables, grains, and oils [31,32,33,34,35]. Yang et al. [31] demonstrated that tea polyphenols effectively delayed the degradation of flavor and color difference, and increased TBARs, TVB-N, and pH, resulting in effective inhibition of microbial growth, thus, extending the shelf life of turtles by 3–6 days compared to the blank group.

Nisin is a polypeptide produced by lactic acid bacterial fermentation. Nisin can kill Gram-positive bacteria and bacterial spores but cannot kill Gram-negative bacteria, yeast, mold, and other fungi [36]. Nisin is enzymatically digested by the human proteases; hence, it is non-toxic to the human body and leaves behind no residue [37]. Furthermore, nisin is stable at low temperatures and is resistant to high temperatures and acid [38]. Therefore, it is used globally in the food preservation industry [39,40,41,42,43,44]. In a previous study, nisin effectively inhibited bacterial growth in camel meat [39]. Furthermore, nisin effectively inhibited the bacterial growth in lean pork and prolonged its storage period [44]. The combination of nisin, tea polyphenols, and chitosan extends the shelf life of chilled mutton [45].

Although a single natural preservative can inhibit microorganisms, the inhabitation of THE microbial range, temperature range, pH range, and other conditions in which it will be stable and effective is limited. Hence, in this study, chitosan, tea polyphenols, and nisin—three preservatives—were combined to explore the effects of different types of compound preservatives on the preservation of plant-based meat. We analyzed the sensory evaluation, color difference, pH, TBARS, and the total plate count of *E. coli*, *S. aureus*, and *Salmonella*, indicators of the biological preservative-treated plant-based meat, to reveal the optimum preservative combination and concentration for the storage of plant-based meat products.

## 2. Materials and Methods

### 2.1. Materials

The materials used in this study, along with their sources, were as follows: soybean protein isolate (Shandong Yuwang Ecological Food Industry Co., Ltd., Dezhou, China); pea protein (Yantai Shuangta Food Co., Ltd., Yantai, China); wheat gluten (Fanxian Huanghe Industrial Co., Ltd., Fanxian, China); L-Cysteine (Henan Wanbang Industrial Co., Ltd., Zhengzhou, China); chitosan (deacetylation = 92%, molecular weight = 4.0 × 10^5^), tea polyphenols, and nisin (food grade, Henan Wanbang Industrial Co., Ltd., Zhengzhou, China); double distilled water (Shanghai Yuanye Bio-Technology Co., Ltd., Shanghai, China); trichloroacetic acid (Tianjin Beilian Fine Chemical Development Co., Ltd., Tianjin, China); thiobarbituric acid (TBA) and ethylenediaminetetraacetic acid disodium salt (Shenyang Dongxing Chemical Plan, Shenyang, China); sodium chloride (Tianjin Zhiyuan Chemical Reagent Co., Ltd., Tianjin, China); glucose (Tianjin Damao Chemical Reagent Factory, Tianjin, China); casein tryptone, agar, yeast, violet red bile agar (VRBA), brilliant green lactose bile broth (BGLB), and Rappaport Vassiliadis soya broth (RVS broth) (Beijing Aoboxing Biology Technology Co., Ltd., Beijing, China); chromogenic *S. aureus* Agar (Guangzhou Huankai Biotechnology Co., Ltd., Guangzhou, China); and chromogenic *Salmonella* agar (Qingdao Haike Biotechnology Co., Ltd., Qingdao, China); vacuum bags (Shijiazhuang Xilong Packaging Co., Ltd., Shijiazhuang, China). All chemicals used were of an analytical grade, and all food products were food grade.

### 2.2. Plant-Based Meat Production and Treatment with Preservatives

**Figure 1:**
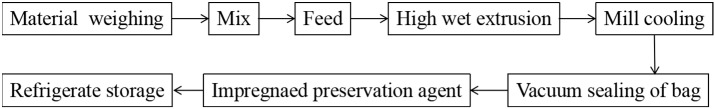
Process flow.

The plant-based meat made in our laboratory comprised soybean protein isolate (4 kg), wheat gluten (7 kg), pea protein (9 kg), l-cysteine (1 kg), and rice bran protein (0.01 kg). The process flow and extrusion operation parameters are shown in Figure 1.

The operational key points are as follows: (1) according to the formula ratio, the different materials were weighed and poured into the mixer to mix the components; (2) the extrusion parameters to make the plant-based meat were as follows: extrusion temperature: 160 °C, screw speed: 280 r/min, solid addition: 8.5 kg/h, water addition: 13.5 kg/h; (3) within the sterile chamber, the samples were cut, put into different combinations of preservative solutions, impregnated for 5 min, and drained; (4) the drained samples were then packed in a vacuum bag, ultraviolet sterilized, and stored in the refrigerator at 4 °C.

The optimal treatment concentrations of the three preservatives were determined by a single factor test, followed by recombination of the three components. The combination of composite preservative was 1% CS + 2.5% TPs, 1% CS + 0.04% nisin, 2.5% TPs + 0.04% nisin, and 1% CS + 2.5% TPs + 0.04% nisin. The index changes of plant-based meat products were determined on days 0, 7, 14, 28, 42, 56, and 70.

**Figure 2:**
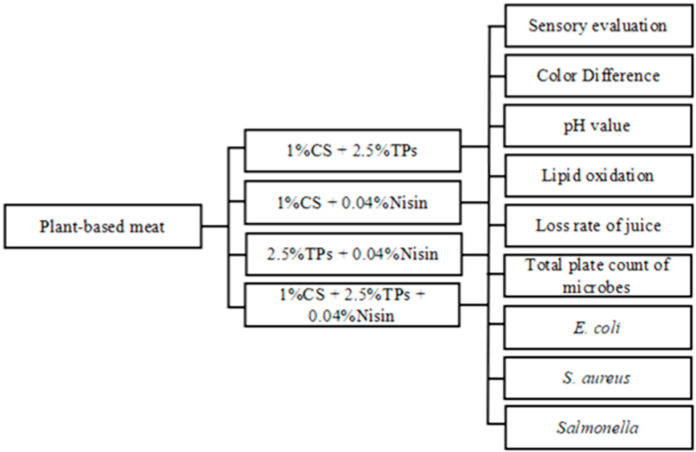
Experimental flow.

Ultraviolet sterilization: Plant-based meat samples were tiled under the UV lamp and turned on to turn the samples every hour. After 4 h of irradiation, the samples were refrigerated at 4 °C. The experimental flow is shown in Figure 2.

### 2.3. Sensory Evaluation

The sensory scoring group, which defined the sensory attributes, consisted of four males and six females. The scoring rules are summarized in Table 1. Members of the sensory scoring panel were selected according to GB/T 10220-2012 and ISO 6658: 2005. (1) Having the general ability to engage in sensory analysis tasks, including the particular sensitivity to the examined stimuli. (2) Initiative (will and interest). (3) Good health (no history of special allergies or medication) and good hygiene. The experimental procedure of the sensory scoring was slightly modified from GB/T 13868-2009 and ISO 8589-2007. Plant-based meat was removed from the freezer. The outer packaging of the test samples should be opened and placed on the sensory scoring operation table according to the serial number. Sensory team members should not eat or be exposed to stimulating food or items before judgment; they score plant-based meat according to their preferences and sensory scoring table.

### 2.4. Determination of Color Difference

L* (Luminosity), a* (redness), and b* (yellowness) values were determined on preprocessed 5 cm × 5 cm × 0.5 cm meat samples using the DP-400 chroma meter (Konica Minolta, Inc., Tokyo, Japan).

### 2.5. Determination of pH Value

The pH values of the plant-based meat products were determined according to the National Standard of China (GB 5009.237-2016). The plant-based meat samples (5 g) were minced with a homogenizer and dissolved in 45 mL of double-distilled water. After shaking the sample at 550× *g* for thirty minutes, the Beckman Avanti J-E centrifuge (Beckman Coulter Commercial Enterprise Co., Ltd., Brea, CA, USA) was centrifuged at 14,700× *g* for 20 min. The pH of the supernatant was determined using an ED-1 pH meter (Shanghai Leici Chuangyi Apparatus & Instrument Co., Ltd., Shanghai, China). Each sample was measured three times, and the results were averaged.

### 2.6. Determination of Lipid Oxidation

The fat content of the plant-based meat was determined with slight modifications according to Chinese national standards GB 5009.6-2016 and ISO 1443-1973. Plant-based meat is hydrolyzed with concentrated hydrochloric acid, then extracted with petroleum ether, and finally evaporated by the rotary evaporator. The resulting substance is the total fat content of the plant-based meat. We determined the fat content of the plant-based meat used in the experiment; the specific results were 5.1%. TBARS values, measures of secondary oxidation products in meat, are commonly used to assess the oxidative rancidity of lipids. TBARS indicate the extent of unsaturated fatty acids oxidized in food and are measured by the malonaldehyde content. Malonaldehyde, under acidic and high temperatures, can react with thiobarbituric acid (TBA) to produce a reddish-brown substance with a maximum absorption wavelength of 532 nm.

The TBARS of plant-based meat were determined according to the National Standard of China (GB 5009.181-2016). We added 5 g of plant-based meat into a 100 mL plug conical bottle, added 50 mL of 0.5 mol/L trichloroacetic acid mixture, shook the mixture, and sealed the bottle. The samples were placed on a constant temperature oscillator (Shanghai Shanzhi Instruments Equipment Co., Ltd., Shanghai, China) of 50 °C at 550× *g* for 30 min. They were cooled to room temperature and filtered. Next, 5 mL of the filtrate was placed in a 25 mL plug color tube, 5 mL of the trichloroacetic acid mixture as the sample blank, and 5 mL of 0.02 mol/L TBA were added, stuffed, mixed, placed in a 90 °C water bath for 30 min, and cooled to room temperature. The absorbance value of the sample solution was measured by adjusting the sample blank at 532 nm, and the malondialdehyde content was obtained according to the standard curve (y = 5.3725x − 0.0184, R^2^ = 0.999) and the calculation Formula (1).

The calculation formula for the malondialdehyde content is as follows:

(1) $$X=\frac{c\times V\times 1000}{m\times 1000},$$

where X is the malondialdehyde content in the samples, unit: mg/kg; c is the concentration of malondialdehyde obtained from the standard curve, unit: µg/mL; V is the fixed volume of the sample solution, unit: mL; m is the sample quality represented by the final sample solution, unit: g; 1000 is the conversion coefficient.

### 2.7. Loss Rate of Juice

The loss rate of juice (X) was calculated as follows:

(2) $$X=\frac{m_{2}-{\;m}_{1}-{\;m}_{0}}{m_{2}-{\;m}_{1}}\times 100\%,$$

where m_2_ is the packed plant-based meat product weight, m_0_ is the bag weight, and m_1_ is the dry weight of meat (obtained by opening the packaging and using a filter paper to absorb the liquid on the surface of the plant-based meat mass).

### 2.8. Microbial Analysis

Microbial tests were performed slightly modified according to the methods of GB (GB 4789.2-2016; GB 4789.3-2016; GB 4789.10-2016), AOAC (AOAC 2000.07), and ISO (ISO 4833-2003; ISO 6887-1-1999; ISO 6888-1-1999). The preservative effects on the total plate count of microbes, *E. coli*, *S. aureus*, and *Salmonella* of plant-based meat were studied using plate count agar. First, approximately 25 g of the sample was mixed with 225 mL of 0.9% stroke-physiological saline solution in a sterile stomacher. The mixture was homogenized by a sterile homogenizer (LC-PJ-400GM, Shanghai Lichen Instrument Technology Co., Ltd., Shanghai, China). Next, the uniform mixture was diluted using 0.9% of stroke-physiological saline solution by ten-fold serials.

(1) A total of 1 mL of the suspension was injected onto an agar plate medium. The inoculated plates were placed in an incubator at 37 °C. After one day of incubation, the total viable counts were measured.
(2) A total of 1 mL of the suspension was injected onto VRBA. The inoculated plates were placed in an incubator at 37 °C. From the VRBA medium, a plate with 15–150 suspected *E. coli* colonies was selected after a day of incubation. Ten colonies were picked into ten tubes with BGLB medium from the plate to observe gas production and record the number of gas-producing tubes. The proportion of gas-producing tubes was multiplied by the number of counted plate colonies, and the dilution was the number of coliform colonies per gram sample.
(3) To determine *S. aureus*, the bacterial suspension was prepared from the plant-based meat and 1 mL of the suspension was injected onto chromogenic *S. aureus* agar and cultured for 24 h before the number of colonies in red was recorded.
(4) Similarly, for *Salmonella*, the plant-based meat broth suspension was pre-enriched and enriched, seeded from the RVS broth onto chromogenic *Salmonella* media, and cultured for 24 h before it was observed whether red colonies were produced.

All operations were performed on a sterile operating table. The results are expressed as lg colony forming units (CFU) per gram of meat.

### 2.9. Statistical Analysis

A complete randomized block design was used in our experiments. The analysis of the statistical variance test analyzed the data, and significant differences were determined using Tukey’s test (at *p* < 0.05), using Origin 2019b (IBM, Chicago, IL, USA). Each experiment was carried out in triplicate, and all data are denoted as mean ± standard error.

## 3. Results and Discussion

### 3.1. Sensory Evaluation

**Figure 3:**
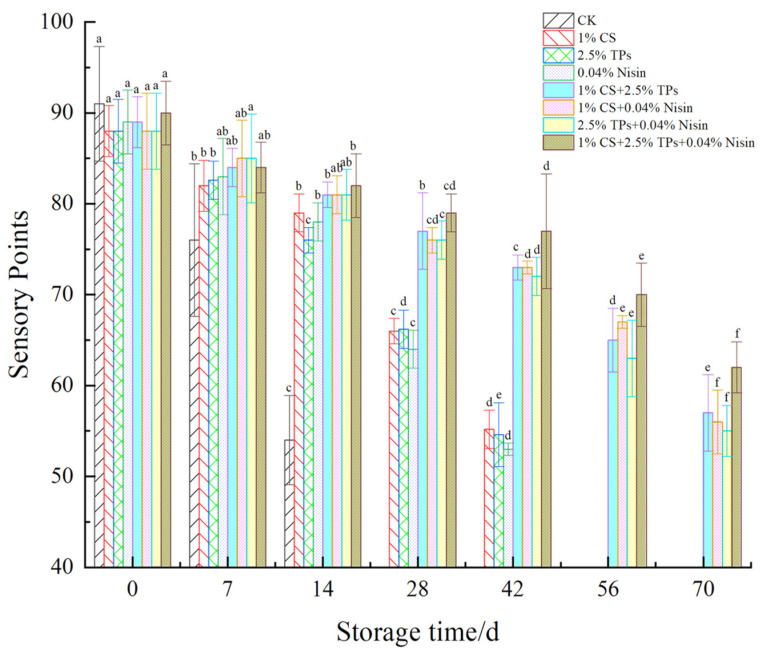
Effects of compound preservatives on the sensory scores of plant-based meat. ^a–f^ Values with different superscripts in the same preservative indicate significant differences (*p* < 0.05). CS, chitosan; TP, tea polyphenols; CK, control group.

The changes in the plant-based meat sensory evaluation are shown in Figure 3. The sensory score of the blank control samples was reduced from 91 to 54 points after 14 days of storage. The sample lost its original taste and had a sour taste, and its texture was soft and loose. The sensory score of plant-based meat treated with preservatives decreased from 90 to 56 points, the color darkened from pale yellow, and the original luster was lost. However, no mildew was observed. Furthermore, the meat had poor elasticity; its fiber structure loosened and had an acidic taste after the storage period. Plant-based meat is markedly different from the beef and lamb studied by Gedarawatte [28] and Pabast [44] in terms of odor and hardness. The odor changes in plant-based meat can be clearly perceived by the sensory scoring groups, and the hardness of plant-based meat gradually decreased. These changes may be attributed to the increase in microorganisms, the utilization of plant-based meat nutrients, and the elimination of metabolic waste, resulting in the deterioration of plant-based meat. Sensory quality decreased in the experimental group as compared to the control group. Blank control samples exhibited significant differences within 14 days; however, no differences were observed within 28 days compared to the meat treated with the compound preservatives, showing that bio-preservatives could maintain the sensory quality of plant-based meat. The three preservatives for composite-treated samples had higher sensory scores.

### 3.2. Color Difference

**Figure 4:**
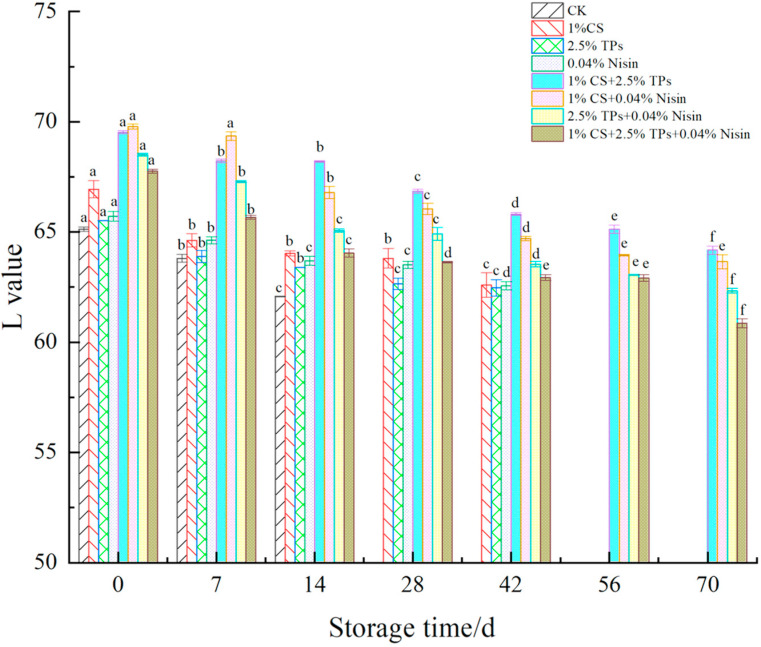
Effects of compound preservatives on the “L value” of plant-based meat. ^a–f^ Values with different superscripts in the same preservative indicate significant differences (*p* < 0.05). CS, chitosan; TP, tea polyphenols; CK, control group.

**Figure 5:**
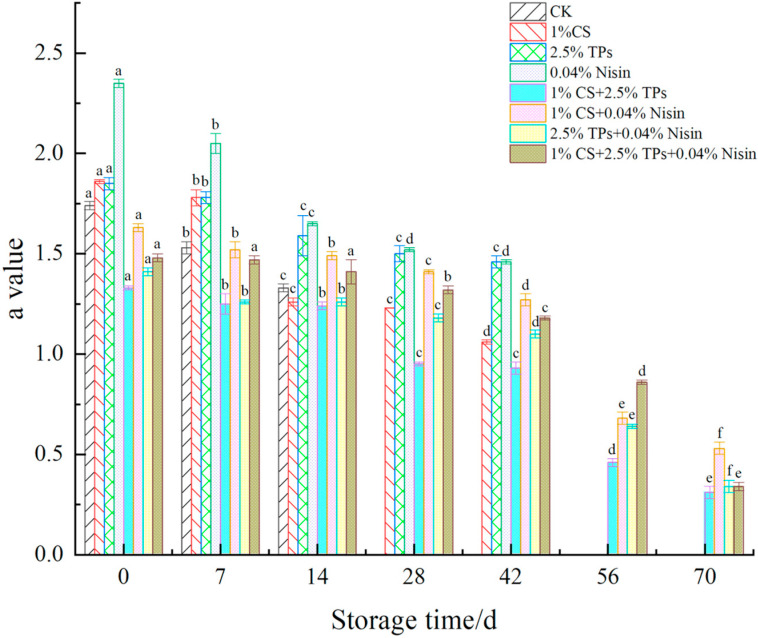
Effects of compound preservatives on the “a value” of plant-based meat. ^a–f^ Values with different superscripts in the same preservative indicate significant differences (*p* < 0.05). CS, chitosan; TP, tea polyphenols; CK, control group.

**Figure 6:**
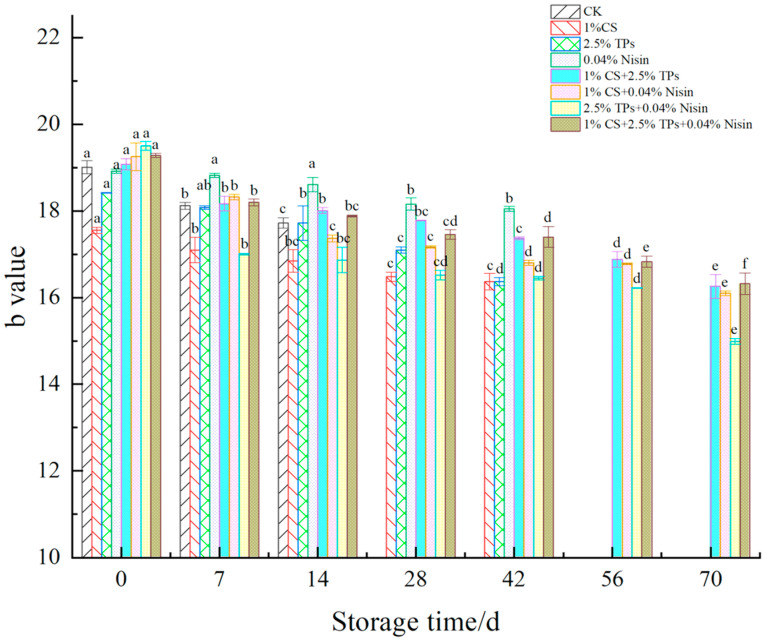
Effects of compound preservatives on the “b value” of plant-based meat. ^a–f^ Values with different superscripts in the same preservative indicate significant differences (*p* < 0.05). CS, chitosan; TP, tea polyphenols; CK, control group.

The color difference is the most direct indicator of a reaction in food. The changes in the color difference of plant-based meat are shown in Figure 4, Figure 5 and Figure 6. After preservative treatment, the L*, a*, and b* values of plant-based meat increased. The preservatives effectively delayed color changes in plant-based meat compared to the controls. The L*, a*, and b* values decreased with the increase in the color difference of plant-based meat. The L*, a*, and b* values of preservative-treated plant-based meat samples decreased from approximately 69 to 62, 1.6 to 0.3, and 19 to 16, respectively, suggesting that the color of plant-based meat deepened. The color of plant-based meat gradually changed from pale yellow to gray–white, losing its luster. Lower values may be associated with lipid oxidation [45]. During storage, L*, a*, and b* values were significantly different across all experimental groups. The variation in color difference of plant-based meat is consistent with changes in animal meat, such as pork [29] and chicken [46]. The difference is that plant-based meat shows higher L* and b* values, likely due to the raw material. The ingredients for making plant-based meat are all white or yellow powdered substances. After extrusion, the plant-based meat is pale yellow. Compared with animal meat, the reason for the lower a* value may be that the plant-based meat is simulated and it lacks red cells and hemoglobin [47].

### 3.3. pH Value

The effects of compound preservatives on the pH of plant-based meat are presented in Table 2. The pH of plant-based meat improved with composite preservative treatment. However, the pH decreased, and after 70 days of storage, the pH of the plant-based meat treated with chitosan and tea polyphenols or with chitosan, tea polyphenols, and nisin decreased to approximately 6. Treatment with composite preservatives delayed the decrease of the plant-based meat pH compared to the treatment with single preservatives. This is consistent with changes in animal meat products during pre-storage. The difference is that the pH value of animal meat products will rise in the later storage period. The reason is that the glycolytic enzyme is blunted and the residual glycogen increases the pH [48]. The trend regarding the pH of plant-based meat is consistent with soy products [49]. The pH decrease may be due to the production of lactic acid by yeast and lactic acid bacteria or the oxidation of vitamins [50]. The pH of partially-preserved plant-based meat increased due to microbes breaking down the protein in the meat product to release amido (-NH_2_), causing higher pH values [51]. The difference in pH between the 1% chitosan and 0.04% nisin treatment and the control was less significant during the 70 days of storage. However, the remaining treatment groups had significant differences in their pH compared to the control. The use of bio-preservatives slowed the reduction of pH in plant-based meat due to the antimicrobial activity of bio-preservatives toward various microorganisms, including fungi, yeast, and bacteria.

### 3.4. Lipid Oxidation

The compound preservative effects of plant-based meat on TBARS are presented in Table 3. TBARS for composite and single preservative treatments exhibited an increase followed by a decrease. This showed the same change as Khan [52] who studied beef lipid oxidation. The current findings are in agreement with the results previously reported [53,54]. However, the peak of TBARS of the compound preservatives compared to a single preservative was delayed. The decrease in malonaldehyde may be due to the production of tertiary lipid oxides [55]. The surge of TBARS occurred during storage due to reactive oxygen species during lipid oxidation, accelerating the oxidation process [56]. There were insignificant changes in TBARS between the two- and three-preservative treatments of the plant-based meat samples. However, the TBARS in the early and late storage periods varied significantly between the meat samples treated with compound preservatives. It was found that 1% chitosan and 0.04% nisin prevented the oxidation of lipids in plant-based meat more effectively.

### 3.5. Loss Rate of Juice

Although the main component of the lost fluid is water, soluble proteins, inorganic salts, vitamins, and other extracts are also lost [57]. The loss of the components reduces the weight and affects the flavor and nutritional value of the food. The effect of compound preservatives on the juice turnover rate of plant-based meat is presented in Table 4. The loss rate of juice of the plant-based meat treated with single and compound preservatives increased with preservation time. This is consistent with pork preservation [58], where lower sap turnover improves the water retention of meat products [59]. The plant-based meat treated with the three composite preservatives only lost 3.97% of juice after 70 days of storage. The increase in the juice turnover rate with storage time was due to metabolic waste generated by microbial growth and reproduction. In addition, the drying of the meat samples resulted in the outflow of the residual water, causing the loss of juice. Treatment with the preservative combinations containing chitosan had a lower rate of juice loss than the non-chitosan-containing preservatives due to the film-forming property of chitosan. A protective film formation on the plant-based meat surface reduced the juice loss. All experimental groups varied significantly in the rate of juice loss during the storage period.

### 3.6. Microbial Analysis

**Figure 7:**
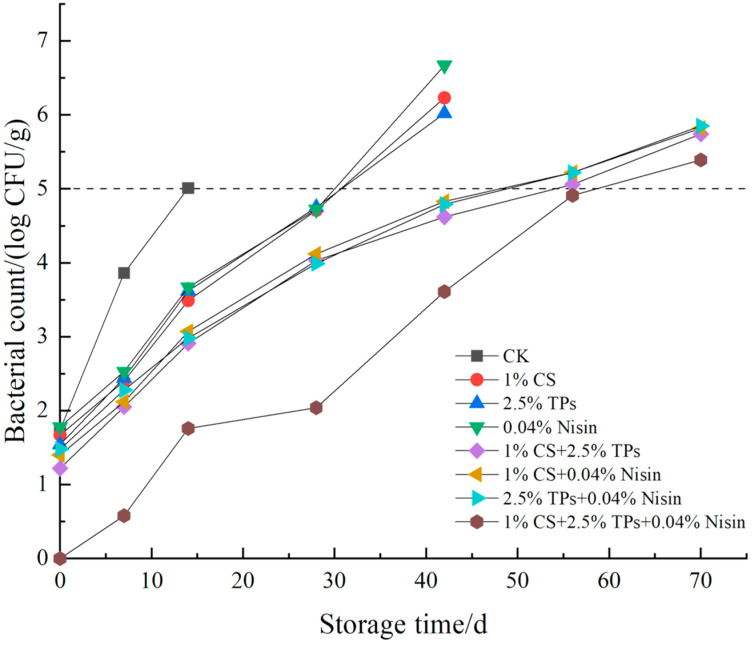
Effects of the compound preservatives on the total bacterial count of plant-based meat. CFU, colony-forming units; CS, chitosan; TP, tea polyphenols; CK, control group.

**Figure 8:**
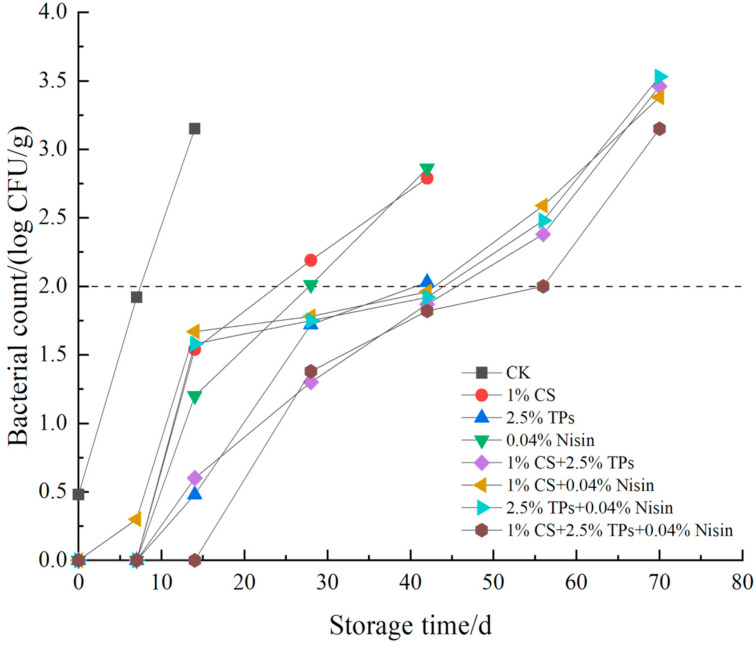
Effects of compound preservatives on *E. coli* of plant-based meat. CFU, colony-forming units; CS, chitosan; TP, tea polyphenols; CK, control group.

**Figure 9:**
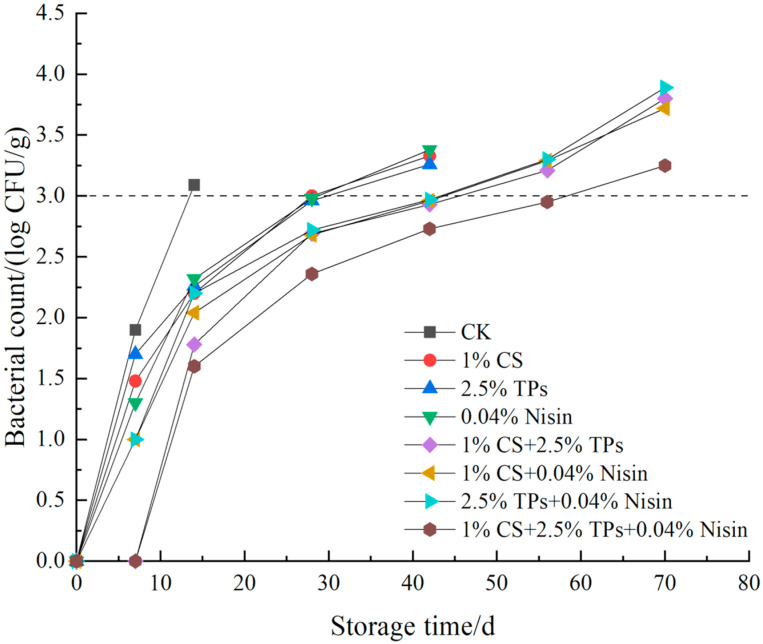
Effects of compound preservatives on *S. aureus* of plant-based meat. CFU, colony-forming units; CS, chitosan; TP, tea polyphenols; CK, control group.

The microbial indicators for the total plate count of microbes, *E. coli*, and *S. aureus* are shown in Figure 7, Figure 8 and Figure 9, respectively. For the blank control sample at 14 days, the total plate count of microbes, *E. coli*, and the *S. aureus* exceeded 5, 2, and 3 lg CFU/g, respectively, which was more than the threshold recommended by the National Standard of China (GB 2726-2016, T/CIFST 001-2020). Thus, it can be inferred that the shelf life of the blank control samples was approximately 14 days. Furthermore, the inhibition effect of compound preservatives on the microorganisms was better than that of a single preservative. The total plate counts of microbes, *E. coli*, and *S. aureus* exceeded the threshold set by the National Standard of China at 56 days after the treatment of plant-based meat with two kinds of preservatives. However, the microbial count of the samples treated with the three-preservative combination did not exceed the set threshold at 56 days, but was near the national standard provisions. The microbial index of plant-based meat with two kinds of preservatives exceeded the national standard in 42 days. The microbial index of plant-based meat with three kinds of preservatives exceeded the national standard in 56 days. Throughout the experiment, the existence of *Salmonella* was not detected; the national standard recommends that *Salmonella* not be detected in plant-based meat, indicating that the laboratory self-made plant-based meat met the production requirements. Thus, overall, the biological preservatives can indeed extend the shelf life of plant-based meat. The three-preservative compound treatment had the best effect on prolonging the shelf life of the samples. Chitosan and tea polyphenols can inhibit most microorganisms, and nisin can significantly inhibit Gram-positive bacteria. Three preservative compound treatments extending the shelf life of foods were also reported previously [60]. The initial amount of plant-based meat is less than meat products [61,62], probably because plant-based meat is squeezed at a high temperature, which kills the microbes in the raw powder.

## 4. Conclusions

This study investigated the combinational effects of chitosan, tea polyphenols, and nisin on the preservation of plant-based meat. When the vacuum-packed blank control sample was stored for 14 days, the tissue structure became loose and the samples became sour. The total microbial plate count and the number of *E. coli* and *S. aureus* exceeded the national standard provisions of China’s requirements, indicating the deterioration of the plant-based meat. The meat samples treated with a two-preservative combination reached the national standard threshold for deterioration at 42 days. Thus, the shelf life of the meat extended from 14 to 42 days compared to the meat samples without preservatives. However, the plant-based meat samples treated with the combination of 1% chitosan, 2.5% tea polyphenols, and 0.04% nisin had the best preservation effect. The shelf life of the samples treated with the preservative was four times (56 days) longer than that of the blank control samples (14 days).

The addition of preservatives delayed the change in the quality of plant-based meat and extended its shelf life. The use of preservatives effectively delays the change of the sensory score and the color change of plant-based meat; it effectively prevents the change of the pH value, fat oxidation, and juice loss rate of plant-based meat. Thus, the compound preservative can be effective in the plant-based meat industry. This study focused on microbial growth changes and explored the effects of compound preservatives on the shelf life of plant-based meat. The shelf life of plant-based meat was approximately determined. In the future, microbial growth prediction models should be established by shortening the experimental cycle to determine the more accurate shelf life of plant-based meat and supplement relevant physical and chemical experiments, such as TVB-N and structural properties.

## Figures and Tables

**Table 1:** Sensory scoring table.

Score Metrics	Specific Rules	Scores
Color (20 points)	pale yellow, shiny, and mildew free	13–20 points
	pale yellow, with lost luster and mildew free	7–12 points
	grayish or darker yellow with mildew	0–6 points
Form (35 points)	great elasticity, moderate hardness, with obvious meat fiber, no mucus	27–35 points
	good elasticity, too hard or too soft, without obvious meat fiber, no mucus	14–16 points
	poor elasticity, unacceptable hardness, no meat fiber, mucus	0–13 points
Odor (35 points)	strong fragrance of specific products, no odor, no acidic taste	27–35 points
	fragrance of a specific product, no odor, no acidic taste	14–16 points
	weak fiber, smelly, sour	0–13 points
Impurities (10 points)	no impurities visible to the naked eye	10 points

**Table 2:** Effects of compound preservatives on the pH of plant-based meat.

Preservation Types	Storage Time/d						
	0	7	14	28	42	56	70
CK	6.52 ± 0.00 ^a^	6.34 ± 0.00 ^b^	6.11 ± 0.00 ^c^				
1% CS	6.67 ± 0.01 ^a^	6.51 ± 0.00 ^b^	6.48 ± 0.01 ^bc^	6.40 ± 0.00 ^d^	6.45 ± 0.06 ^cd^		
2.5% TPs	6.42 ± 0.00 ^a^	6.28 ± 0.00 ^c^	6.28 ± 0.00 ^c^	6.39 ± 0.00 ^b^	6.23 ± 0.00 ^d^		-
0.04% Nisin	6.65 ± 0.00 ^a^	6.57 ± 0.00 ^b^	6.38 ± 0.00 ^c^	6.22 ± 0.00 ^d^	6.21 ± 0.03 ^d^		
1% CS + 2.5% TPs	7.47 ± 0.01 ^a^	6.97 ± 0.00 ^b^	6.90 ± 0.00 ^b^	6.81 ± 0.00 ^c^	6.65 ± 0.00 ^d^	6.51 ± 0.00 ^e^	6.07 ± 0.00 ^f^
1% CS + 0.04% Nisin	7.48 ± 0.00 ^a^	7.42 ± 0.03 ^ab^	7.36 ± 0.00 ^b^	6.50 ± 0.01 ^c^	6.59 ± 0.09 ^c^	6.37 ± 0.00 ^d^	6.29 ± 0.00 ^d^
2.5% TPs + 0.04% Nisin	7.35 ± 0.01 ^a^	6.92 ± 0.02 ^b^	6.86 ± 0.00 ^c^	6.73 ± 0.01 ^d^	6.46 ± 0.00 ^e^	6.31 ± 0.01 ^f^	6.17 ± 0.01 ^g^
1% CS + 2.5% TPs + 0.04% Nisin	7.45 ± 0.00 ^a^	6.82 ± 0.00 ^b^	6.80 ± 0.01 ^b^	6.58 ± 0.06 ^c^	6.49 ± 0.00 ^d^	6.37 ± 0.02 ^e^	6.10 ± 0.04 ^f^

^a–g^ Different letters in the same line mean statistically significant differences by the Tukey’s test (*p* < 0.05). CS, chitosan; TP, tea polyphenols; CK, control group.

**Table 3:** Effects of compound preservatives on TBARS of plant-based meat.

Preservation Types	Storage Time/d						
	0	7	14	28	42	56	70
CK	0.000 ± 0.000 ^c^	0.232 ± 0.013 ^b^	0.268 ± 0.011 ^a^				
1% CS	0.000 ± 0.000 ^e^	0.018 ± 0.000 ^d^	0.132 ± 0.000 ^c^	0.160 ± 0.001 ^b^	0.170 ± 0.000 ^a^		
2.5% TPs	0.102 ± 0.001 ^e^	0.126 ± 0.002 ^d^	0.243 ± 0.001 ^b^	0.498 ± 0.000 ^a^	0.208 ± 0.002 ^c^		
0.04% Nisin	0.098 ± 0.000 ^e^	0.122 ± 0.000 ^d^	0.265 ± 0.000 ^c^	0.324 ± 0.000 ^a^	0.322 ± 0.004 ^b^		
1% CS + 2.5% TPs	0.083 ± 0.029 ^e^	0.221 ± 0.045 ^d^	0.257 ± 0.011 ^cd^	0.290 ± 0.000 ^c^	0.490 ± 0.017 ^a^	0.367 ± 0.000 ^b^	0.319 ± 0.013 ^bc^
1% CS + 0.04% Nisin	0.002 ± 0.004 ^d^	0.030 ± 0.046 ^cd^	0.126 ± 0.095 ^bc^	0.152 ± 0.013 ^b^	0.261 ± 0.027 ^a^	0.188 ± 0.006 ^ab^	0.188 ± 0.006 ^ab^
2.5% TPs + 0.04% Nisin	0.000 ± 0.000 ^e^	0.019 ± 0.027 ^e^	0.126 ± 0.039 ^d^	0.243 ± 0.013 ^c^	0.352 ± 0.013 ^a^	0.327 ± 0.004 ^ab^	0.283 ± 0.013 ^bc^
1% CS + 2.5% TPs + 0.04% Nisin	0.000 ± 0.000 ^d^	0.011 ± 0.010 ^d^	0.035 ± 0.013 ^d^	0.146 ± 0.019 ^c^	0.250 ± 0.025 ^b^	0.381 ± 0.006 ^a^	0.241 ± 0.025 ^b^

^a–e^ Different letters in the same line mean statistically significant differences by the Tukey’s test (*p* < 0.05); Unit: mg/kg. CS, chitosan; TP, tea polyphenols; CK, control group.

**Table 4:** Changes in the turnover of plant-based meat juices treated with compound preservatives.

Preservation Types	Storage Time/d						
	0	7	14	28	42	56	70
CK	-	1.99 ± 0.77 ^b^	5.16 ± 1.47 ^a^				
1% CS	-	0.38 ± 0.07 ^d^	1.78 ± 0.70 ^c^	2.71 ± 0.28 ^b^	3.47 ± 0.14 ^a^		
2.5% TPs	-	0.71 ± 0.07 ^d^	1.10 ± 0.21 ^c^	1.92 ± 0.84 ^b^	3.61 ± 0.56 ^a^		
0.04% Nisin	-	0.58 ± 0.21 ^d^	1.85 ± 0.42 ^c^	2.98 ± 0.14 ^b^	4.53 ± 0.49 ^a^		
1% CS + 2.5% TPs	-	0.04 ± 0.00 ^f^	0.51 ± 0.21 ^e^	1.14 ± 0.63 ^d^	2.73 ± 0.56 ^c^	3.26 ± 0.91 ^b^	5.55 ± 1.33 ^a^
1% CS + 0.04% Nisin	-	0.23 ± 0.14 ^f^	0.66 ± 0.49 ^e^	1.43 ± 0.63 ^d^	2.98 ± 0.28 ^c^	3.65 ± 0.63 ^b^	5.93 ± 0.91 ^a^
2.5% TPs + 0.04% Nisin	-	0.31 ± 0.21 ^f^	0.81 ± 0.42 ^e^	1.59 ± 0.70 ^d^	3.15 ± 0.84 ^c^	3.89 ± 0.56 ^b^	5.06 ± 0.91 ^a^
1% CS + 2.5% TPs + 0.04% Nisin	-	0.02 ± 0.00 ^e^	0.30 ± 0.07 ^e^	0.97 ± 0.35 ^d^	1.48 ± 0.28 ^c^	2.83 ± 0.84 ^b^	3.97 ± 1.05 ^a^

^a–f^ Different letters in the same line mean significant statistical differences by Tukey’s test (*p* < 0.05); Unit: %. CS, chitosan; TP, tea polyphenols; CK, control group.

## Data Availability

The datasets generated for this study are available upon request to the corresponding author.
